# MicroRNAs can regulate human APP levels

**DOI:** 10.1186/1750-1326-3-10

**Published:** 2008-08-06

**Authors:** Neha Patel, David Hoang, Nathan Miller, Sara Ansaloni, Qihong Huang, Jack T Rogers, Jeremy C Lee, Aleister J Saunders

**Affiliations:** 1 Department of Bioscience & Biotechnology, Drexel University, Philadelphia, PA, USA; 2 Wistar Institute, Philadelphia, PA, USA; 3 Neurochemistry Laboratory, Massachusetts General Hospital, Harvard Medical School, Boston, MA, USA; 4 Department of Molecular, Cell, & Developmental Biology, University of California, Santa Cruz, CA, USA; 5 Departments of Biochemistry & Molecular Biology and Neurobiology & Anatomy, Drexel University College of Medicine, Philadelphia, PA, USA

## Abstract

A number of studies have shown that increased APP levels, resulting from either a genomic locus duplication or alteration in *APP *regulatory sequences, can lead to development of early-onset dementias, including Alzheimer's disease (AD). Therefore, understanding how APP levels are regulated could provide valuable insight into the genetic basis of AD and illuminate novel therapeutic avenues for AD. Here we test the hypothesis that APP protein levels can be regulated by miRNAs, evolutionarily conserved small noncoding RNA molecules that play an important role in regulating gene expression. Utilizing human cell lines, we demonstrate that miRNAs hsa-mir-106a and hsa-mir-520c bind to their predicted target sequences in the *APP *3'UTR and negatively regulate reporter gene expression. Over-expression of these miRNAs, but not control miRNAs, results in translational repression of *APP *mRNA and significantly reduces APP protein levels. These results are the first to demonstrate that levels of human APP can be regulated by miRNAs.

## Results

Accumulating evidence suggests that increased expression of the amyloid precursor protein gene (*APP*) increases Alzheimer's disease (AD) risk. The resulting increase in APP protein levels results in increased Aβ levels, leading to synaptic dysfunction, neurodegeneration and, eventually, cognitive decline.

APP levels can be regulated at the genomic, transcriptional or translational level. At the genomic level, Down's Syndrome (Trisomy 21) patients have three copies of the *APP *gene and develop AD symptoms early in life [1]. Similarly, duplication of the *APP *locus, in the absence of a full trisomy 21, also leads to early-onset AD [2]. Dysregulation of *APP *transcription can also increase the risk of AD. Genetic variants in the *APP *promoter increase *APP *transcription by ~2–3 fold and have been reported to increase AD risk [3]. Growth factors have been reported to control *APP *mRNA half-life [4]. These growth factors effects are dependent on a 29 bp sequence in the *APP *3' UTR [4,5]. APP translation is also regulated; for example, IL-1 can induce an increase in APP translation [6]. IL-1 is a pro-inflammatory cytokine and genetic variants have been linked to increased AD risk [7,8]. Taken together, these findings provide strong evidence that increased APP levels increase AD risk.

MicroRNAs (miRNAs) are small noncoding RNAs that control gene expression post-transcriptionally. Complementary binding between miRNAs and sequences within the 3' UTR of target genes results in repression of target gene expression by translational inhibition or mRNA degradation [9]. Approximately 700 miRNA genes are encoded in the human genome and recent evidence demonstrates that some miRNAs are differentially expressed in AD patients compared to age-matched controls [10]. These differences in miRNA expression may play an important role in AD pathogenesis. In an attempt to address this possibility, we test the hypothesis that miRNAs can regulate APP levels.

**Figure 1:**
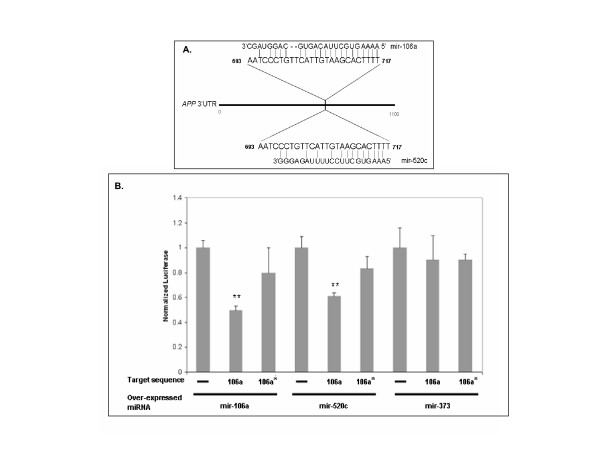
**mir-106a target sequence regulates reporter gene expression**. **(A) **Predicted mir-106a and mir-520c target sites in the 3'UTR of APP. **(B) **Over-expression of mir-106a or mir-520c, but not mir-373, significantly reduced luciferase expression (p = 0.0006) controlled by the putative mir-106a *APP 3'*UTR target sequence. This reduction is not observed when a seed region mutant of mir-106a (106a*) is utilized. For all experiments, three independent trials were performed. Error bars represent standard deviation. *p < 0.05; **p < 0.01, compared to the appropriate control.

Bioinformatic analysis predicts that the 3' UTR of human *APP *contains 28 unique miRNA target sites [11,12]. To experimentally confirm that APP levels can be regulated by miRNAs, we chose to initially study miRNA hsa-mir-106a (mir-106a; Figure 1A) since (i) the putative target site in the *APP *3'UTR is 100% complementary to the seed region of the miRNA, (ii) it has a large free energy of seed region binding, and (iii) it is expressed in human brain [13]. To determine if the putative mir-106a target site in the *APP *3'UTR is capable of regulating gene expression, we cloned it into the 3' UTR of firefly luciferase. We co-transfected this reporter into naïve HEK-293 cells along with a mir-106a over-expression vector [14] and measured luciferase activity (Figure 1B). We observed a significant ~50% decrease (p < 0.0001) in luciferase activity when the putative mir-106a target site was included in the reporter compared to either a reporter lacking the putative target site or reporter carrying a seed-region mutant of the putative mir-106a target site. To determine if this effect was simply due to over-expressing miRNAs, we repeated the experiment while over-expressing mir-373, a miRNA not predicted to target the *APP *3'UTR. We observed no change in luciferase activity. Another miRNA, mir-520c, shares the same seed region target sequence as mir-106a but is not expressed in human brain (Figure 1A) [13]. Therefore we tested mir-520c, we observed that mir-520c over-expression significantly decreased luciferase activity when the putative mir-106a target site was included in the reporter compared to either a reporter lacking the putative target site or a reporter carrying a seed-region mutant of the putative mir-106a target site (Figure 1B). We repeated these experiments in the human neuroblastoma cell line SH-SY5Y and observed similar results (data not shown). To confirm that the miRNAs were being over-expressed, we utilized RT-QPCR to quantify miRNA levels and observed significant increases in both mir106a and mir-520c (Table 1) levels (p < 0.0001).

**Table 1:** Relative miRNA levels

	**Fold Change 2^-ΔΔCt^**	**p-value**
	
**Vector**	1	
**mir-106a**	30.1 ± 1.2	< 0.0001
**mir-520c**	1964.6 ± 1.1	< 0.0001

QPCR results demonstrate a significant increase in mir-106a and mir-520c levels in response to over-expression compared to cells transfected with an empty vector.

**Figure 2:**
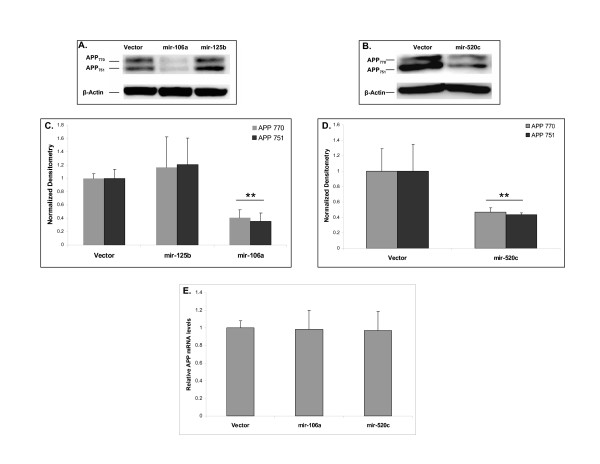
**mir-106a and mir-520c can regulate APP levels post-transcriptionally**. APP_770 _and APP_751 _levels are reduced in cells over-expressing **(A) **mir-106a compared to cells expressing either mir-125b or the empty vector and **(B) **mir-520c compared to cells expressing the empty vector. **(C) **Quantification of these Western blot results reveals that mir-106a over-expression significantly decreases APP levels (p < 0.01) compared to cells expressing mir-125b or cells transfected with the empty vector. **(D) **Quantification of these Western blot results reveals that mir-520c over-expression significantly decreases APP levels (p < 0.01) compared to cells transfected with the empty vector. **(E) **QPCR results show that *APP *mRNA levels are not altered by mir-106a or mir-520c over-expression. For all experiments, three independent trials were performed. Error bars represent standard deviation. *p < 0.05; **p < 0.01, compared to the appropriate control.

Having demonstrated that over-expression of mir-106a or mir-520c was capable of repressing reporter gene expression via interaction with its putative target site, we investigated whether over-expression of these miRNAs could decrease endogenous APP levels in human cell lines. We transfected naïve HEK-293 with mir-106a and mir-125b over-expression vectors and then performed quantitative Western blot analysis to determine APP steady state levels. We utilized mir-125b as a negative control since it is not predicted to target APP but has increased expression in AD brain [10]. We observed that mir-106a over-expression significantly decreased APP levels (Figure 2A). Both APP isoforms expressed in this cell line, APP_770 _and APP_751_, were significantly and similarly affected. Mir-106a over-expression reduced APP levels by ~50% (p < 0.01) compared to cells transfected with the empty vector (Figure 2C). Over-expression of mir-125b had no significant effect on APP levels. Over-expression of mir-520c had a similar effect on APP levels as mir-106a (Figure 2B and 2D).

Most human miRNAs repress gene expression by inhibiting translation and do not affect target gene mRNA levels [15,16]. This seems to be the case in our experimental setting. We utilized RT-QPCR to determine if miRNA over-expression resulted in decreased *APP *mRNA levels. Over-expression of mir-106a or mir-520c had no effect on *APP *mRNA levels (Figure 2E). Mir-106a and mir-520c, therefore, appear to inhibit translation of the *APP *transcript.

Our results are the first to experimentally demonstrate that human APP levels can be regulated by miRNAs. In 2004, it was predicted that APP levels could be regulated by miRNAs [12]; recently it was shown that expression of the *C. elegans *orthologue of *APP*, *APL-1*, is regulated by developmentally-timed miRNAs [17].

In human neurons the APP_695 _isoform is the predominant expressed isoform. All APP isoforms (APP_695_, APP_751 _and APP_770_) share the same 3' UTR [18] therefore we expect that the mir-106a mediated regulation of APP levels that we observe in the HEK-293 cell line should also occur in neurons given that mir-106a is expressed in the brain. We do not expect mir-520c mediated APP regulation to occur in neurons since this miRNA is not expressed in brain. It is important to test if miRNA regulation is a normal aspect of APP metabolism in neurons. If so, it will be important to determine whether AD pathogenesis is affected by alterations in miRNA function and/or expression. It is possible that aging- or environment-induced changes in miRNA expression, and/or sequence variation in miRNAs or their targets, contribute to increased APP levels and increased AD risk. Recently, it was demonstrated that expression of the β-secretase BACE can be regulated by miR-29a/b-1 and mir-107; furthermore, increased BACE levels correlated with decreased miR-29a/b-1 and mir-107 levels in AD patients [19,20].

Regardless of the biological roles of miRNA in APP metabolism, therapeutics based on miRNA-induced decrease in APP levels would offer a treatment targeting the underlying pathophysiology of the disease. In the near future, substantial progress will be made in understanding the role of miRNAs in AD pathogenesis and in therapeutic approaches to treating AD.

## Methods

### Reporter Vectors and DNA constructs

Reporter vectors containing the putative miRNA target sites from the *APP 3'*UTR, were synthesized with double-stranded oligos perfectly complementary to putative miRNA target sites and oligos in which the seed regions were mutated. The mir-106a target oligos had the sequence (seed region bolded):

5' CTAGTAATCCCTGTTCATTGTAA**GCACTTT**TGCTCAGCA 3'

3' ATTAGGGACAAGTAACATT**CGTGAAA**ACGAGTCGTTCGA 5'

The mutant mir-106a target oligos had nucleotides three through six of the seed region mutated (italicized):

5' CTAGTAATCCCTGTTCATTGTAA**GC*GTCC*T**TGCTCAGCA 3'

3' ATTAGGGACAAGTAACATT**CG*CAGG*A**ACGAGTCGTTCGA 5'

We utilized established methods [21] to clone these synthetic versions of putative miRNA target sites into a luciferase reporter gene (pMIR-REPORT; Ambion).

### Cells and Cell Culture

Naïve human embryonic kidney (HEK)-293 cells were purchased from ATCC. Cells were cultured in Dulbecco's modified Eagle's medium (DMEM) supplemented with 10% fetal bovine serum, 2 mM L-glutamine,100 units/ml penicillin and 100 μg/ml streptomycin.

### Transfections and Luciferase Assays

10,000 Naïve HEK-293 were plated in 24 well plates. The next day, cells were transfected with a miRNA overexpression vector [14], reporter vectors bearing either the miRNA target sequence or the miRNA seed region mutant target sequence, and one tenth of the molar volume of pRL-SV40, a Renilla Luciferase control vector. We utilized Arrest-In transfection reagent (Open Biosystems Inc.); any differences in transfection efficiency were accounted for by measuring Renilla luciferase activity. 48 hours post-transfection, cell were lysed using 100 μL of GLB (Glo Lysis Buffer, Promega). Firefly and Renilla luciferase activities were measured using a dual luciferase reporter assay kit (Promega), per the manufacturer's protocol. Firefly luciferase activity was normalized to Renilla luciferase activity.

### Western Blot Analysis

200,000 Naïve 293 cells were plated in 6 well plates. The next day, cells were transfected with a miRNA overexpression vector. Using previously described methods [22], quantitative Western blots were performed using equal amounts of total protein.

### Antibodies

A polyclonal antibody specific for the C-terminus of human APP (A8717; Sigma Aldrich, Inc) and a monoclonal antibody specific for human β-Actin (A5441; Sigma Aldrich) were used as primary antibodies. Secondary antibodies were HRP-conjugated goat anti-rabbit (GE Healthcare) and HRP-conjugated goat anti-mouse (GE Healthcare).

### RNA extraction and Quantitative PCR

48 hours post-transfection, cells were washed with cold PBS and total RNA was isolated using RNeasy Mini Kit (Qiagen Inc.). To quantify APP mRNA levels, cDNA was synthesized using total RNA, N6 random primers and SuperScript II Reverse Transcriptase (Invitrogen). cDNA was then diluted 1:15 using RNase free water and mixed with APP or GAPDH primer/probe sets (Applied Biosystems, Inc.; APP Catalog # Hs00169098_m1; GAPDH Catalog # Hs99999905_m1), 2× PCR Universal Master Mix (Applied Biosystems, Inc.) and amplified using an ABI 7500 Real Time PCR system following the manufacturer's directions. GAPDH was used as an internal control. To determine differences in *APP *mRNA levels, we utilized the ΔΔCt method.

To quantify miRNA levels, cDNA was reverse transcribed from total RNA samples using specific miRNA primers from the TaqMan MicroRNA Assays and reagents from the Taq Man MicroRNA Reverse Transcription kit (Applied Biosystems). The resulting cDNA was amplified by PCR using TaqMan MicroRNA Assay primers with the TaqMan Universal PCR Master Mix and analyzed with a 7500 ABI PRISM Sequence Detector System (Applied Biosystems) according to the manufacturer's instructions. The relative levels of miRNA expression were calculated from the relevant signals by the ΔΔCt method by normalization to the signal of RNU44 [23].

### Statistical Analysis

Values in the text and figures are presented as means ± standard deviations of experiments carried out in triplicate, at least. Each experiment was carried three times. Equal variance or separate variance two-sample student's t-test were used, as appropriate, to compare two groups. Where appropriate, Bonferroni analysis was used to correct for multiple comparisons within a single experiment.

## Competing interests

AJS declares that he is a share holder in TorreyPines Therapeutics. The remaining authors declare that they have no competing interests.

## Authors' contributions

All authors have read and approved the final manuscript. NP designed the experiment, acquired, analyzed and interpreted the data and drafted the manuscript. DH and NM cloned the target and mutant sequences in the reporter vectors. SA helped to draft and edit the manuscript. JTR contributed towards experimental design. QH contributed towards experimental design and provided miRNA over-expression vectors.JCL performed the statistical analysis. AJS oversaw the experimental design, data analysis, data interpretation, and drafting/editing the manuscript.
